# Supplementary material for: Granzyme K initiates IL-6 and IL-8 release from epithelial cells by activating protease-activated receptor 2
Source: PLoS One. 2022 Jul 26;17(7):e0270584. doi: 10.1371/journal.pone.0270584 (PMC9321427; doi:10.1371/journal.pone.0270584)
Supplement: Attachment — Submitted filename: Response to Reviewers.docx [file pone.0270584.s002.docx]

Reviewers Comments

Major:

1. Introduction: Some key citations are missing eg. p4: “GzmK cleaves and activates PAR1 on endothelial cells and fibroblasts (Sharma et al., 2016; Turner et al., 2019)” should be amended to include Cooper et al., 2011. This should also be included in first line of Results “Previous studies have indicated that GzmA and GzmK can cleave and activate PARs (Suidan et al., 1994; Sower et al., 1996b; Sharma et al., 2016) given its relevance to the present article. Recent articles in Immunity, 2021 suggesting a role for GzmK in inflammaging as well as the recent paper citing a role for extracellular GzmB in maternally-transferred childhood asthma citing a mechanism involving PAR2 published in J Clin Invest (Qian et al, 2020) should also be mentioned possibly in the intro and/or discussion.

The Cooper et al and Qian et al references have been included. The inflammaging paper (Immunity 2021) was already referenced in the discussion (Ref 32).

2. It is unclear as to why GzmB-mediated PAR2 activation was not explored given the high impact study published last year in the Journal of Clinical Investigation by Qian et al suggesting a key role for extracellular GzmB-mediated PAR2 activation of airway epithelial cells as the mechanism as to how maternal exposure to diesel exhaust particles predispose offspring to allergic airway disease.

Granzyme B experiments were performed prior to the publication of the Qian et al paper and we are not in a position to perform them any more. As such, we have included references to the Qian paper and removed references to GzmB not cleaving PAR2.

3. Results are well-described although it is unclear as to why GzmB was dropped after PAR1. GzmB was also shown to induce IL-8 release via an unknown mechanism (Hiroyasu et al, Nature Comm, 2021). Given that GzmB was not assessed throughout the manuscript, it may be worth focussing exclusively on GzmA and K and dropping the GzmB data as it is incomplete. Alternatively, adding GzmB throughout for PAR2/IL6/8 studies could be added to give a more complete picture. The latter would require substantial revision.

The primary focus of this paper was GzmA and GzmK. GzmB was used in the luciferase assays as a control for cleavage away from the consensus site. We have made this more clear in the text.

4. Discussion Line 3 states “GzmA and GzmK cleave both PAR1 and PAR2, while GzmB cleaves only PAR1.” This contradicts the study by Qian et al (J Clin Invest, 2020) in which a substantial amount of evidence would suggest otherwise in a clinically-relevant model. This must be supported by data and a strong discussion if disputing this work given the impact. I would suggest removing the GzmB PAR2 work and any text dismissing GzmB-mediated PAR2 activation as this was not adequately assessed in the present manuscript.

As described above, we have altered our references to GzmB and more clearly defined its role as a control in the earlier experiments.

Minor

1. Methods: Please include number of replicates where relevant.

This has been included

2. Results: ‘Cleavage of full-length PAR1 and PAR2 by GzmA and GzmK’: Literature reports a sharp decrease in activity of the NanoLuc system below pH 8, whereas Gzms optimally function around pH 7. A brief mention of the potential limitations should be discussed.

We disagree that the pH sensitivity of the reagents affects our analyses. All assays were performed in buffered medium at an optimal granzyme cleavage pH (7.4). As strong responses were observed for GzmK, the poor responses for GzmA and GzmB are due to poor cleavage of the protein substrate rather than pH incompatibilities of the system.

3. Discussion: “In lung epithelial cells, GzmK treatment leads to the release of proinflammatory cytokines IL-6 and IL-8, but not TNFα or IL-1β, via a PAR2 and MEK-dependent mechanism.” Include reference.

Data deleted.

4. Discussion: “GzmK treatment leads to the release of proinflammatory cytokines IL-6 and IL-8, but not TNFα or IL-1β, …” is inconsistent with the previous results section which mentioned GzmK treatment did not lead to IFN-y or IL-1β release (TNFα not mentioned). This sentence also mentioned that the mechanisms of IL-6 and IL-8 release were both PAR2 and MEK-dependent, however, the previous results section (and corresponding figure) only investigated this mechanism in the context of IL-8. It should be mentioned that GzmB can induce IL-8 release from keratinocytes (Hiroyasu et al, Nature Comm., 2021)

This lead in paragraph is a brief summary of our results. The wording has been improved.

5. Figures, while one can interpret the results, are of low resolution/blurry in the PDF version.

High resolution images for publication included.

6. Figures, 1a/b: Describe source of WT PARS. I may have missed where it was discussed in the text.

Described in the methods as coming from Prof Hollenberg (U Calgary)

7. Figures, 2c/d: The experiment group trypsin + buffer is coloured grey instead of green as depicted in the legend.

The figure legend was incorrect. It has been fixed.

8. Figures, 2-5: There are no statistics/p values included in the graphs despite significance values having been mentioned within the figure legends.

P values included on graphs.

9. Figures 3: Since the same experimental groups are presented as in Figure 2, it would be ideal to keep colours/patterns consistent for treatments.

This has been done.

10. Figure 4: The figure legend is inconsistent with the presented images – there is no (c) or (d). As a result, the labels are also incorrect. There was no display of IL-8 release shown.

This has been fixed.

11. Figure 5: For clarity for the reader, include in brackets in the figure legend the identity of each PAR inhibitor, as done in text. Also, there was no mention in the figure legend of the MEKi (PD184352) or what the solid bars represent (treatment with GzmK vs activating peptide). Previous figures used the nomenclature GzmK-AI instead of AEBSF. For consistency, it should be the same.

This has been fixed.

Editors Comments

1. Please ensure that your manuscript meets PLOS ONE's style requirements, including those for file naming. The PLOS ONE style templates can be found at

https://journals.plos.org/plosone/s/file?id=wjVg/PLOSOne_formatting_sample_main_body.pdf and

https://journals.plos.org/plosone/s/file?id=ba62/PLOSOne_formatting_sample_title_authors_affiliations.pdf

The text file has been reformatted.

2. Please review your reference list to ensure that it is complete and correct. If you have cited papers that have been retracted, please include the rationale for doing so in the manuscript text, or remove these references and replace them with relevant current references. Any changes to the reference list should be mentioned in the rebuttal letter that accompanies your revised manuscript. If you need to cite a retracted article, indicate the article’s retracted status in the References list and also include a citation and full reference for the retraction notice.

The reference list is correct.

3. We note that the grant information you provided in the ‘Funding Information’ and ‘Financial Disclosure’ sections do not match.

When you resubmit, please ensure that you provide the correct grant numbers for the awards you received for your study in the ‘Funding Information’ section.

4. Thank you for stating the following in the Acknowledgments Section of your manuscript:

"This work was supported by the National Health and Medical Research Council Australia (Project Grant 1141421 and Program Grant 490900), National Institutes of Health (NS102722, DE026806, DK118971, DE029951) and Department of Defense (W81XWH1810431)."

We note that you have provided funding information that is not currently declared in your Funding Statement. However, funding information should not appear in the Acknowledgments section or other areas of your manuscript. We will only publish funding information present in the Funding Statement section of the online submission form.

Please remove any funding-related text from the manuscript and let us know how you would like to update your Funding Statement. Currently, your Funding Statement reads as follows:

"This work was funded by a National Health and Medical Research Council Australia Project Grant 1141421 (PIB & AS), a National Health and Medical Research Council Australia Program Grant 490900 (PIB), National Institutes of Health grants NS102722, DE026806, DK118971 and DE029951 (NB) and Department of Defense grant W81XWH1810431 (NB). The funders had no role in study design, data collection and analysis, decision to publish, or preparation of the manuscript."

Please include your amended statements within your cover letter; we will change the online submission form on your behalf.

The funding information has been removed from the manuscript. I cannot find our original funding information, however I can confirm that the funding statement is correct.

5. In your Data Availability statement, you have not specified where the minimal data set underlying the results described in your manuscript can be found. PLOS defines a study's minimal data set as the underlying data used to reach the conclusions drawn in the manuscript and any additional data required to replicate the reported study findings in their entirety. All PLOS journals require that the minimal data set be made fully available. For more information about our data policy, please see http://journals.plos.org/plosone/s/data-availability.

Upon re-submitting your revised manuscript, please upload your study’s minimal underlying data set as either Supporting Information files or to a stable, public repository and include the relevant URLs, DOIs, or accession numbers within your revised cover letter. For a list of acceptable repositories, please see http://journals.plos.org/plosone/s/data-availability#loc-recommended-repositories. Any potentially identifying patient information must be fully anonymized.

Important: If there are ethical or legal restrictions to sharing your data publicly, please explain these restrictions in detail. Please see our guidelines for more information on what we consider unacceptable restrictions to publicly sharing data: http://journals.plos.org/plosone/s/data-availability#loc-unacceptable-data-access-restrictions. Note that it is not acceptable for the authors to be the sole named individuals responsible for ensuring data access.

We will update your Data Availability statement to reflect the information you provide in your cover letter.

The underlying primary data has been provided as a supplementary file.

6. Please amend the manuscript submission data (via Edit Submission) to include author Andrea Leong.

Andrea has been added.

7. We note that you have included the phrase “data not shown” in your manuscript. Unfortunately, this does not meet our data sharing requirements. PLOS does not permit references to inaccessible data. We require that authors provide all relevant data within the paper, Supporting Information files, or in an acceptable, public repository. Please add a citation to support this phrase or upload the data that corresponds with these findings to a stable repository (such as Figshare or Dryad) and provide and URLs, DOIs, or accession numbers that may be used to access these data. Or, if the data are not a core part of the research being presented in your study, we ask that you remove the phrase that refers to these data.

The reference to data not shown has been removed.
